# Getting In and Out of Mitosis*

**DOI:** 10.5041/RMMJ.10051

**Published:** 2011-07-31

**Authors:** Tim Hunt

**Affiliations:** Nobel Laureate in Physiology or Medicine, 2001; Cancer Research UK, London Research Institute, London, UK.

## INTRODUCTION

Mitosis is the process of one cell dividing into two replicate cells. This process is a universal one, occurring throughout the phyla. Mitosis is a fundamental process. Without it, life ceases to exist.

A series of preparatory steps must take place before a cell enters mitosis, and these steps must be undone to exit mitosis. The cell has a nucleus which contains the genetic material, the DNA, as chromosomes packaged with proteins. Before the cell undergoes mitosis, the cell must make two copies of its DNA so that each daughter cell has a complete copy of the genetic material. At the onset of mitosis the nuclear envelope dissolves, and each chromosome lines up with its copy. The chromosomes are connected to proteins known as the mitotic spindle, which in turn are connected to two centrosomes at the two opposite poles of the cell. At the climax of mitosis, the two chromosome copies are pulled apart and each copy is assigned to opposite daughter cells. This is how cell inheritance works. The information on how to direct these dividing cells into making a human being or any organism is contained in those chromosomes. The whole point of cell division is to distribute the DNA molecules (the chromosomes) to each daughter cell accurately and equally. When that goes wrong and a cell has lost genetic material or has received a surplus of genetic material, the result may be cell death, a defective cell, or cancer.

During mitosis, under light microscopy, it seems at first that nothing is happening. Suddenly, the cell splits into two daughter cells followed by another period of quiescence. It is as if a switch is turned on and then turned off again. An on/off mechanical switch, like a light switch, may look very simple from the outside, but it has many intricate parts inside. So too, the biological switch which controls mitosis has very intricate inner workings. This article explores these inner workings.

## ENTERING MITOSIS

**Figure 1:**
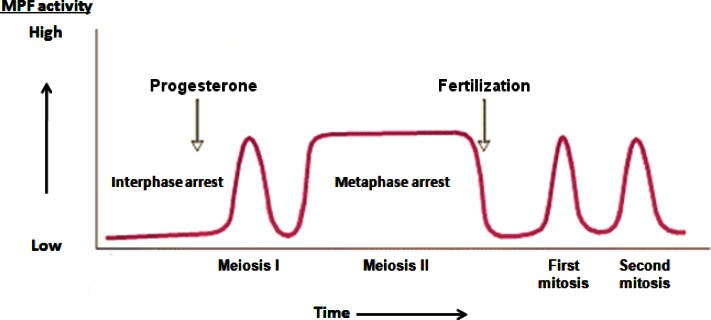
Oscillation of maturation promoting factor (MPF) activity during meiotic and mitotic cell cycles of *Xenopus* oocytes and early frog embryos.

The first clue as to how cells enter mitosis came in the early 1970s from studying reproductive physiology in frogs. When progesterone is added to an oocyte, it takes on a bi-polar appearance: half of the oocyte turns black, and the other half turns white. There is also a white dot in the middle of the black hemisphere. Looking at the white dot under a microscope, one sees the chromosomes lined up in the middle, bound to the mitotic spindle. It was later found that progesterone turns on an enzyme that catalyzes cell division. When this protein is turned on, it catalyzes the breakdown of the nuclear envelope, the alignment of the chromosomes, the assembly of the spindle, and the separation to two daughter cells. It was also discovered that this enzyme has a positive feedback loop; activation of this enzyme led to further activation of this enzyme. This protein was dubbed MPF or maturation-promoting factor. It took an additional 15 years to purify MPF, but during this time the properties of this protein were revealed. MPF levels were high during cell division, but during S-phase, when the DNA was replicated, or in interphase, when the cells were growing in size, MPF could not be detected (Figure 1). This appearing, disappearing, and reappearing was true for frog egg fertilization and for subsequent cell divisions. MPF was also found in star-fish, having the same characteristics of frog MPF. A decade later, MPF was discovered in humans as well. These MPFs were interchangeable. When star-fish MPF was put into frog cells, it caused those cells to divide.

In the early 1980s, I became interested in the control of protein synthesis in sea-urchin eggs. Eggs were taken at different time points after fertilization, and their proteins were run on a one-dimensional SDS polyacrylamide gel (SDS-PAGE). This gel separates the proteins by their molecular size, each protein showing up as a band on the gel. A certain band on the gel appeared and disappeared in time with the cell division cycle in a way that was very reminiscent of the behavior of MPF. This was a very important discovery because the band disappeared at specific times in the cell cycle. In a later experiment using clam eggs, we observed two such bands that appeared and disappeared at specific times during the cell cycle. These two proteins were dubbed cyclin A and B, since their appearance cycled during the cell cycle and at the time I was also an avid cyclist. If the cell cycle is blocked by inhibiting chromosomal replication, these proteins do not go away. Cyclin A starts disappearing before cyclin B, but the reason for that is still not known.

Andrew Murray in Marc Kirschner’s lab showed that all that is needed to induce a frog egg to undergo mitosis is the production of cyclin. When the cyclin reaches a certain level, mitosis begins; and after mitosis the cyclin disappears. Murray also showed that if the cyclin constitutively stays on by mutating the protein, the cell is now locked at the height of mitosis, dividing again and again, until eventually the cells die. In short, the cyclin must be produced to enter mitosis and be destroyed to exit mitosis. It was later shown that the cyclins are degraded via the ubiquitin degradation pathway.

## THE CYCLIN–CDK DIMER MODEL

**Figure 2:**
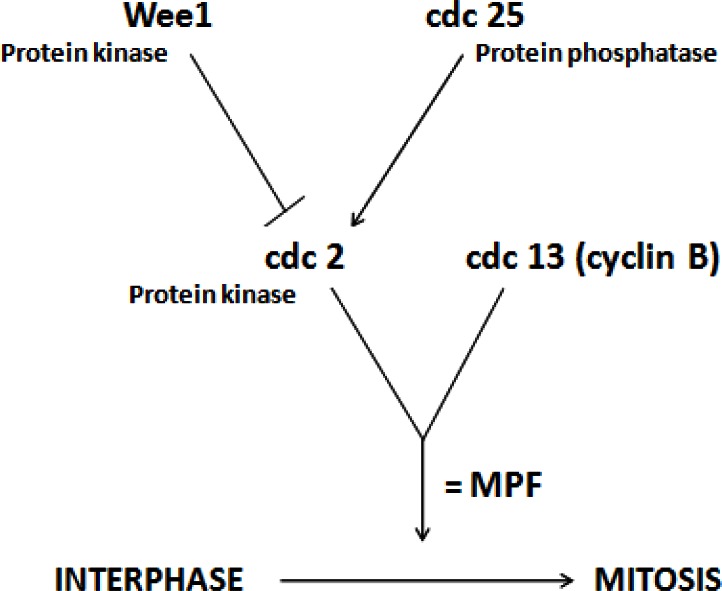
MPF is made up of two proteins, a protein kinase (cdc2 in yeast) whose levels are constant throughout the cell cycle and a cyclin (cdc 13 in yeast) whose levels fluctuate during the cell cycle.

Using a parallel model in yeast, it was shown that MPF was made up of two proteins: one protein that cycled (cdc13 or cyclin B), that is, was present in the cell during certain times of the cell cycle and absent at other times, and another protein (cdc2) that was present throughout the cell cycle (Figure 2). When cyclin binds to cdc2, it changes the conformation of the protein by pulling a loop of the cdc2 protein away, and, along with the attachment of a phosphate group on cdc2, it activates the protein. This protein is a kinase, and it attaches phosphate groups onto its target proteins. The phosphorylation of the kinase causes a small change in the structure of the protein, but that change causes the kinase to be 100-fold more active. The enzyme can be turned off in one of two ways: either by degradation of the cyclin or by the removal of the phosphate group by another enzyme, a phosphatase.

**Figure 3:**
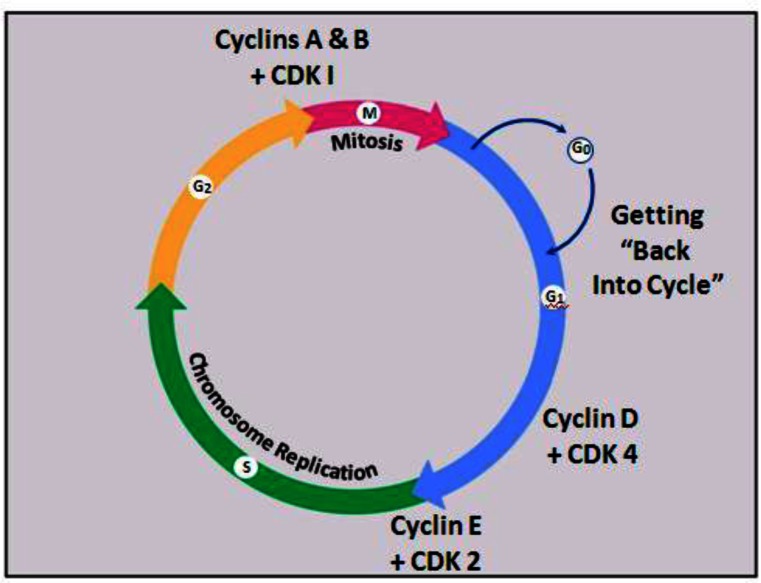
The CDK-cyclin pairing motif is used by the cell as checkpoints at different time points throughout the cell cycle.

The paradigm of having a complex made up of two proteins, one being a protein whose levels cycle throughout the cell cycle (cyclins) and the other a protein whose levels are constant throughout the cell cycle (CDKs or cyclin-dependent kinases), is used not only during mitosis but also during other stages of the cell cycle. To enter mitosis, cyclins A and B bind to CDK1. To enter S-phase, when the chromosomes are replicated, cyclin E and CDK2 are needed. For a quiescent cell to start the whole process and enter the cell cycle, cyclin D and CDK4 must be there (Figure 3).

CDK’s single function is to phosphorylate proteins. With the discovery of these proteins, researchers were busy identifying the target proteins and the levels of phosphorylation needed for these target proteins to induce the cell into the different cell cycle stages.

Purified cyclin A and B were made together with the CDK. The purified proteins, made in bacteria, were shown to phosphorylate proteins when ATP was added to the mix. SDS-PAGE protein gels were run with one lane containing the total proteins from a cell without the kinase and another lane containing the total proteins from a cell with the active kinase. Any protein that was phosphorylated ran a little slower than its non-phosphorylated form. That was the first experimental design used to identify the substrates of cyclin A and B-CDK. About 1% of the proteins showed this phosphorylation shift. Later, when phosphor-proteomic methods were used, a long list of over 100 protein substrates was compiled. However, this list did not explain how the cells entered and exited mitosis. Sidney Brenner called this method of proteomics “low-input, high-throughput, no-output research”, and he was right.

## GETTING OUT OF MITOSIS

If proteins have to be phosphorylated by kinases to become activated and get into mitosis, then it makes perfect sense that these phosphate groups on the activated proteins must be removed by phosphatases in order to become inactivated and get out of mitosis. However, as we shall see, the story is slightly more complicated.

**Figure 4:**
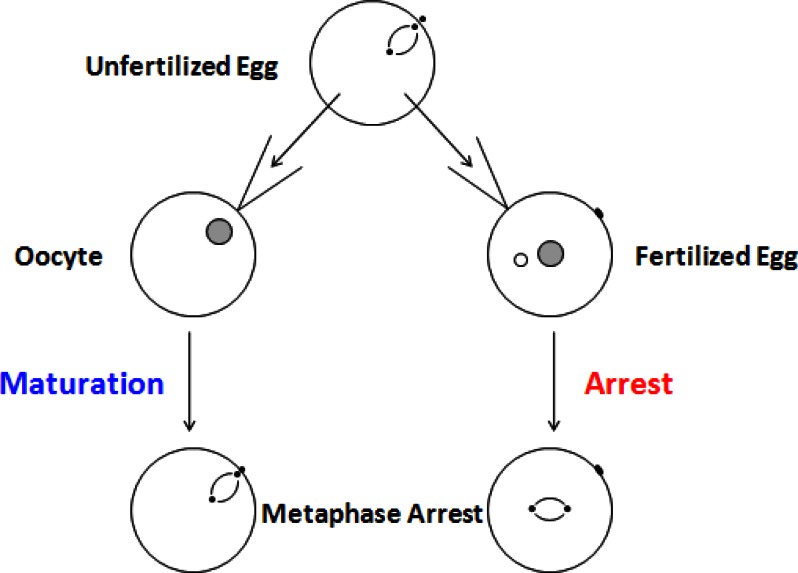
Cytostatic factor (CSF) and maturation promoting factor (MPF) are the same thing and have different effects depending on the developmental stage of the cell.

In frog oocytes, during metaphase, the levels of MPF stay high for an extended period of time. This period is called metaphase arrest (Figure 2). The biological reason behind this arrest is that the egg actually stays in metaphase with the chromosomes all lined up until the sperm arrives. The protein that maintains this arrest was discovered by Mazoui in 1971. It was called cytostatic factor (CSF) because it blocks the progress of the cell cycle. In actuality, there is no difference between MPF and CSF. If cytoplasm is taken out of an unfertilized egg and injected into an oocyte, it initiates the auto-catalytic loop that turns on the formation of cdc2-cyclin (or MPF) and causes an acceleration of the cell cycle. If the same cytoplasm is injected into a fertilized egg which divides on its own accord, the result is a cell cycle arrest (Figure 4). The cell arrests in metaphase with the chromosomes lined up on the mitotic spindle. Since CSF is heat-labile and protease-sensitive, it is clearly a protein.

In 1989, CSF was discovered to be the product of an oncogene normally found only in oocytes called c-MOS. c-MOS, which is a protein kinase, was later discovered to activate other protein kinases. This cascade is now known as the MAP-kinase (MAPK) pathway, whereby c-MOS phosphorylates MEK, MEK phosphorylates MAPK, MAPK phosphorylates p90^RSK^, whose target is Erp1, a protein discovered in 2005. Phosphorylated Erp1 is a potent inhibitor of the degradation of cyclin. As long as Erp1 is phosphorylated, the cell stays in metaphase arrest.

## THE ROLE OF CALCIUM IN EXITING MITOSIS

Once the sperm reaches the oocyte, the metaphase arrest is broken. In all species tested, once the sperm connects with the oocyte, there is a surge in calcium levels within the oocyte which breaks the metaphase arrest and allows the cell to go forward and divide. This happens by activating another kinase called calcium calmodulin-activated kinase II (CaMKII) which then phosphorylates Erp1 on other sites in the protein. CaMKII does not phosphorylate Erp1 directly. It phosphorylates another kinase called Polo which then goes and phosphorylates Erp1. Erp1 is then degraded by the ubiquitin pathway. This allows for the degradation of cyclin B which inactivates the CDK activity, enabling the cell to get out of metaphase arrest and continue dividing.

Proof for the existence of this pathway initiated by calcium was shown by artificially adding calcium to arrested oocytes. When calcium was added, the cyclin was degraded and the cell continued cycling. If a mutated non-calcium-dependent CaMKII protein was added, the same effect was seen.

## THE “ARTIFACT” THAT ROARED

For many years, the above-described model of getting in and out of mitosis had seemed to be complete. However, an experiment by Satoru Mochida complicated this picture. Satoru constructed a mutated form of Erp1 which does not have the sequence that targets Erp1 for destruction via the ubiquitin pathway. When calcium was added to control cells, the cyclins disappeared. When the “indestructible” Erp1 protein was added to the oocytes, even after the addition of calcium, no destruction of cyclin was seen. An additional assay was done, in which the phosphorylation level of one of the subunits of the ubiquitin ligase (APC3) that is responsible for the degradation of the cyclin was measured. When calcium is added to cells, APC3 becomes phosphorylated and when there is no longer any cyclin it becomes dephosphorylated as it should be. However, there is a period of time, after APC3 is phosphorylated, in which it is transiently dephosphorylated and then rephosphorylated again before the destruction of the cyclins. That was a consistent result and not an artifact. The only explanation for this phenomenon is that calcium also activates a phosphatase that dephosphorylates APC3 and, when cellular vesicles take up the calcium and cause free calcium levels to drop, the phosphatase is deactivated, and the kinase rephosphorylates APC3.

Calcium was known to activate a phosphatase called calcineurin, and we demonstrated that calcineurin has a very high and transient peak of activity after the addition of calcium. When cyclosporin A, a specific inhibitor of calcineurin, was added to the cells, there was no dephosphorylation of APC3. However, the degradation of the cyclins was not totally inhibited by the addition of cyclosporin A. Instead, the rate of degradation slowed down. We therefore looked to see whether other proteins were also dephosphorylated with the addition of calcium. When the assay was done on frog oocytes, we saw that there were many proteins that were dephosphorylated with the addition of calcium and their dephosphorylation was blocked when either the mutated Erp1 or CaMKII protein was added to the cells. When cyclosporin A was added to cells, there was a delay of about an hour in getting out of mitosis. When a calcium-independent kinase and phosphatase was added to the cells along with cyclosporin A, this lag in exiting mitosis was abolished.

**Figure 5:**
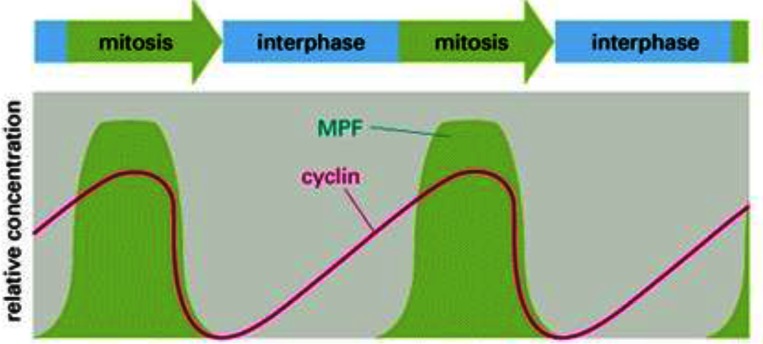
The rise and fall in levels of maturation promoting factor (MPF) and cyclin during the early embryonic cell cycle. (Copyright 1994 from *Molecular Biology of the Cell* by Alberts et al. Reproduced by permission of Garland Science/Taylor & Francis LLC).

Since the oocytes are dependent on calcium to remove the block and allow them to continue dividing, we asked ourselves whether the addition of cyclosporin A blocks somatic cells, which are not calcium-dependent, from dividing. The answer, to our relief, was that calcium is not needed for the normal mitotic cell cycle to happen. However, proteins, such as APC3, were still transiently dephosphorylated. That meant that there were other phosphatases that were turned on during the end of mitosis, since cyclosporin A blocked the phosphatase calcineurin. In addition, there was another question that intrigued us. Before the cell enters mitosis there is a steady elevation of cyclin levels and there is also a linear increase in the activation of CDK. However, when the cells enter mitosis it is not a gradual entry. It is a sudden entry, as if a switch has been turned on (Figure 5). In other words, how do you convert a continuous increase into a sharp on/off behavior?

## PHOSPHATASE X

We noticed that right before the cells enter mitosis there are proteins that become hyper-phosphorylated. We therefore assumed that kinase activity alone is not enough to cause cells to enter mitosis, but there should also be active phosphatases that are turned off. Indeed, such an active phosphatase has been found. It is not induced by calcium, and it is not inhibited by cyclosporin A, but it is inhibited by okadaic acid. When the cell enters mitosis, the activity of this phosphatase is absent, and when the cell exits mitosis, this phosphatase is activated once more. This phosphatase possesses the “switch” properties that we have been looking for. It is turned off to enter mitosis, and it is turned on when the cell exits mitosis.

As of now, we are calling this phosphatase “phosphatase X”, since we do not yet know its identity, nor do we know how it is regulated. The evidence that we have accumulated to date suggests that phosphatase X is a member of the protein phosphatase 2A (PP2A) family. This family of phosphatases has about seven members, and PP2A has around 80 different forms. We know that this phosphatase can be turned on by simply inhibiting CDK activity. This means that the phosphatase itself is controlled by phosphorylation. This also implies that if phosphatase X is negatively controlled by CDK phosphorylation, it must be positively controlled by dephosphorylation, i.e. by yet another phosphatase, phosphatase Y. Another known fact about phosphatase X is that when concentrated cytoplasmic extracts of frog oocytes are diluted, the activity of phosphatase X increases. In a 10× dilution, we find a 100-fold increase in the phosphatase activity, and we do not yet know why.

To summarize, once CDK is activated by binding to a cyclin, it phosphorylates its target proteins. As a result, it activates certain proteins and inhibits either directly or, more probably, indirectly the activity of phosphatase X. This allows the cell to enter mitosis. The cell stays in the mitotic state until the cyclins are degraded and phosphatase X is turned on to allow the cells to exit from the mitotic state and enter interphase. In other words, if you want to fill a sink with water, you have to do two things: turn on the water and put a plug in the drain. If you want to empty the water, the faucet has to be turned off and you must remove the plug as well.

One of the most pleasurable things in science is the voyage into the unknown. We have discovered a control mechanism of mitosis that until recently was totally unknown. There is much left to be discovered in this pathway, such as the identity of the players and their control mechanisms. This voyage of discovery is the beauty of science.

## Abbreviations:

CaMKII: calcium calmodulin-activated kinase II;
CDK: cyclin-dependent kinase;
CSF: cytostatic factor;
DNA: deoxyribonucleic acid;
MPF: maturation promoting factor;
PP2A: protein phosphatase 2A;
SDS-PAGE: sodium dodecyl sulfate polyacrylamide gel electrophoresis.

